# Co-designing interventions to increase food access: perceptions and experiences of community member end-users

**DOI:** 10.1186/s40900-025-00788-y

**Published:** 2025-10-14

**Authors:** Tamara Petresin, Nayssam Shujauddin, Angela Annis, Vicky Drapeau, Jess Haines, f.u.n. Community Advisors

**Affiliations:** 1 Department of Family Relations and Applied Nutrition, University of Guelph, Guelph, Canada; 2 10C Shared Space, Guelph, Canada; 3 Department of Human Health and Nutritional Sciences, University of Guelph, Guelph, Canada; 4 Department of Kinesiology, Université Laval, Québec, Canada

**Keywords:** Co-design, Community engagement, Food access, Community-led interventions

## Abstract

**Background:**

Access to healthy, affordable food is a challenge in many communities. There is growing recognition that co-designed, community-led approaches, which directly involve end-users in the development, implementation, and evaluation processes, are needed to create effective and contextually appropriate food access interventions. However, limited research has examined the experiences and perceptions of the end-users who play a central role in these processes. This study aims to explore the perceptions and experiences of community member end-users (termed Community Advisors) involved in a co-designed food access initiative titled food uniting neighbours (f.u.n.).

**Methods:**

A qualitative approach was used. Data were collected through focus groups and individual interviews with f.u.n. Community Advisors (n = 12).

**Results:**

Four major themes were identified: 1) Motivation to be a Community Advisor, including social connection, helping others, and skill-building; 2) Importance of Community Advisors to the Co-Designed Project, highlighting their role in humanizing the project, building trust, and ensuring community relevance; 3) Facilitators of Community Advisor Success such as mutual respect, teamwork, and administrative support; and 4) Suggestions for Improvement which emphasized the need for greater cultural diversity among the Advisors and more sustainable funding structures.

**Conclusions:**

Community member end-users play a vital role in co-designed food access solutions contributing authenticity, trust, and deep community insight. Findings suggest that inclusive representation, supportive team dynamics, and stable funding may help sustain meaningful engagement of community members in co-designed initiatives. These findings underscore the importance of developing co-designed processes that are not only empowering but also structurally supported to ensure long-term impact.

**Supplementary Information:**

The online version contains supplementary material available at 10.1186/s40900-025-00788-y.

## Abstract (plain language summary)

Access to healthy, affordable food is a serious challenge in many communities. Co-design is a research approach in which people directly affected by an issue help shape the solutions. This approach ensures that programs created reflect the real needs and experiences of the people they are meant to help. In this study, community members with experience facing challenges related to food access co-designed a project, titled food uniting neighbours (f.u.n). These community members, who we refer to as Community Advisors, are local residents who co-run and guide the project with researchers. While co-design is becoming more common, there is limited research on the role of community members and their perceptions of the co-design process. To address this gap, we spoke to 12 Community Advisors through focus groups and interviews to gain insight into why they became involved, the roles they played, and the supports they needed to successfully implement the co-designed project. By understanding their experiences, we can improve how co-design projects are planned, implemented, and supported.

Four key themes emerged:

1. Why they joined—to connect with others, help their community, and build skills.

2. Their impact—they made the project more relatable, trustworthy, and relevant.

3. What helped them succeed—mutual respect, strong teamwork, and good support.

4. What could be better—more cultural diversity among Advisors and stable funding.

The study shows that community members are essential to co-designed projects. To support their involvement, future co-designed projects should prioritize inclusive practices, strong communication, and reliable resources.

**Supplementary Information:**

The online version contains supplementary material available at 10.1186/s40900-025-00788-y.

## Background

Food insecurity, described as "the inability to acquire or consume an adequate diet quality or sufficient quantity of food in socially acceptable ways, or the uncertainty that one will be able to do so" [1] remains a persistent challenge in Canada. Food insecurity rates are at their highest levels nationally, provincially, and in the Wellington-Dufferin-Guelph region, where this project is based [2, 3]. In 2023, 22.9% of Canadians lived in a food-insecure household [3]. More locally, where this project is based, approximately 23% of Wellington-Dufferin-Guelph households were food insecure [2]. Six pillars contribute to food security including availability, access, utilization, agency, sustainability, and overall stability of these pillars [4]. Food access considers the physical, economic, and social resources required to secure food and is an important part of food security [5]. While many initiatives have attempted to address food access, current approaches are insufficient to address this crisis [6–8], and few centre on the voices of those most affected. Many health promotion initiatives take a "top-down" approach where expert opinions develop broad solutions in hopes of meeting the needs of end users [9]. In contrast, co-designed and community-led approaches offer more inclusive and context-sensitive solutions by involving end-users directly in the intervention design process, allowing them the opportunity and space to address contextually specific and complex factors shaping health behaviours [9, 10]. There is growing recognition that co-designed, community-led approaches lead to more effective, contextually appropriate interventions [11]. The food uniting neighbours (f.u.n.) project is an initiative developed in collaboration with community member end-users (termed Community Advisors) and researchers to increase access to nutritious foods using co-design methodology in a low-income neighbourhood in Ontario, Canada. f.u.n. is a new community-led project steered by Community Advisors, faculty and graduate students from the University of Guelph, and staff from 10C, a local community agency that supports grassroots efforts. Broader partners include Wellington-Dufferin-Guelph Public Health and the Guelph Community Health Centre. This project built upon work conducted as part of the Nutritious Food Worksteam of Our Food Future, a multisectoral project led by the City of Guelph/Wellington County from 2020 to 2023. The Nutritious Food Workstream focused on reimagining a food system that is based in equity and dignity by ensuring food security and healthier outcomes for all [12].

Community members play a critical role in co-design processes offering local knowledge, lived experience, and insights that can shape more relevant and responsive interventions [13, 14]. A growing body of literature documents the experiences of diverse partners engaged in participatory research settings, exploring perspectives from researchers, service providers and community members [15, 16]. While some studies may explore the perceptions of community members engaged in participatory research [17, 18], fewer studies focus exclusively on the perspectives of end-user collaborators in relation to how they experience their own involvement in the co-design of research [19, 20]. Understanding community members' experiences can provide valuable insights into the feasibility, acceptability, and perceived impact of co-designed interventions from the perspective of those most closely connected to the communities the interventions aim to serve. This information can inform future co-designed projects by highlighting effective engagement strategies, identifying potential challenges, and guiding best practices for meaningful collaboration. Further, including perceptions of community members supports equity and inclusion by helping to redistribute existing power imbalances often present in community-academic partnerships [21–23] and helps centre historically underrepresented voices in knowledge production [24, 25]. Highlighting the community member experience also demonstrates respect, transparency, and shared ownership, which are critical for ethical and effective community-engaged research [26]. The objective of this paper is to explore the perceptions and experiences of community member end-users (termed Community Advisors) involved in the f.u.n. project to better understand their motivations, roles, and conditions related to being involved in a co-design initiative. Study results will help inform effective community member engagement in future co-designed interventions.

## Methods

### Researcher positionality

This study was informed by a contextual constructivist perspective, which acknowledges that any phenomenon can be understood in multiple ways, with interpretations shaped by the researchers’ perspectives and the specific social context in which the research occurs [27]. T.P., a PhD student and registered dietitian with both quantitative and qualitative nutrition research experience, grew up in Onward Willow and has established relationships with the Community Advisors, conducted the data collection for this study. These connections supported rapport during data collection, while T.P’s training as a health professional likely shaped the lens through which findings were interpreted. The broader author team brought diverse perspectives: N.S., as program lead for f.u.n. with deep community knowledge and practice-based expertise, A.A., V.D., and J.H. are health promotion researchers, with training spanning nutrition, physical activity, and community-based research. Community Advisors, who are also co-authors, contributed their lived experiences and perspectives, which grounded the analysis and challenged purely academic interpretations.

### Research setting

Onward Willow is a vibrant community in Guelph, Ontario, with a population of over 7000 people. The Onward Willow neighbourhood has the highest overall unemployment rate (6.5%) in the Wellington-Dufferin-Guelph region, has a high percentage of families living below the Low-Income Cut-Off (19%), individuals who recently immigrated to Canada (< 5 years; 10%), and single parent families (24%) [28].

### Co-design process

To support the co-design approach, we recruited 13 community members from the Onward Willow neighbourhood as Community Advisors in April 2022 through posters shared at community agencies serving the Onward Willow community, social media posts, and information sessions with members of the research team. Community Advisors were compensated $25 an hour, an amount above the current Guelph living wage of $21.30 an hour [29]. The co-design approach followed the five steps of design thinking proposed by the Hasso-Plattner Institute of Design at Stanford [30]: 1) Empathize and understand community needs/strengths, 2) Define project goals, 3) Ideate interventions to meet goals, 4) Prototype, and 5) Test specific interventions. Throughout the project, Community Advisors collaborated regularly with University of Guelph staff and researchers, as well as personnel from a local community agency supporting local grassroots food access efforts. Together, the f.u.n. team identified food access barriers and mapped existing resources in the community, identified seven potential strategies for improving food access, named the project food uniting neighbours (f.u.n.), and developed a theory of change. Between October and December 2023, the f.u.n. team conducted focus groups with 87 community members to gather feedback [31]. Based on this input, we developed and selected five targeted intervention strategies to improve nutritious food access in Onward Willow for pilot testing. We implemented these five interventions between June and December 2023, and Community Advisors led the interventions with support from staff and researchers who provided guidance and logistical assistance throughout the process. Community Advisors not only helped identify community needs, but also selected intervention strategies, and led the delivery of programs such as food skills workshops and community cafés. Full details regarding the engagement of Community Advisors can be found in Any et al. [31] and full details regarding the development and pilot-testing of interventions to increase food access are reported in Petresin et al. [32].

### Data collection

We invited all 13 Community Advisors to participate in a focus group exploring their perceptions and experience as a Community Advisor. Of those invited, 12 participated in either a focus group or an interview. One Community Advisor declined to participate due to personal circumstances at the time of data collection. We conducted two in-person focus groups ranging from forty minutes to one and a half hours in duration with 11 Community Advisors total, and one in-person interview with one Community Advisor that was thirty minutes long who could not attend either focus group due to scheduling conflicts. Using a semi-structured interview guide (Table 1), we aimed to elicit Community Advisors’ motivations for joining and staying involved with the project, their perceived role and influence in the project, the value they derived from participation, their perception of the effectiveness of the co-design process, and suggestions for improving the co-design process. To help reduce the risk of social desirability bias in the Community Advisors’ responses, we informed them at the start of the focus groups and interview that all feedback, whether positive, neutral, or critical, was welcome and that the goal was to hear all perspectives. It was made clear that any feedback provided would not affect their ongoing involvement with f.u.n. We audio-recorded both focus groups and the interview, and transcribed verbatim for analysis. This manuscript was prepared in accordance with the Guidance for Reporting Involvement of Patients and the Public (GRIPP2) reporting guidelines on patient and public involvement in research [33], a completed GRIPP2 short form checklist can be found in Additional file 1 [see Additional file 1]. All participants were compensated for their time during the focus groups and interview.

**Table 1:** Community Advisor experience focus group questions

1. Why did you want to be a Community Advisor with the f.u.n. Project?
2. What keeps you coming back to the Community Advisor Meetings and the f.u.n. Project?
3. What do you feel you personally get out of being a Community Advisor with the f.u.n. Project?
4. How do you think having Community Advisors adds to the f.u.n. Project?
5. What would be different about f.u.n. if we did not have Community Advisors on the team?
6. What aspects of the f.u.n. Project have you seen the Community Advisors play a role in? Or, in what ways have the Community Advisors influenced how the f.u.n. Project has been implemented?
7. How can we improve the co-design model?
8. What, if any, changes would you recommend to improve the Community Advisor experience?

### Data analysis

Interview transcriptions were imported into Microsoft Word. The focus group and interview qualitative data were analyzed by T.P. using template analysis, a type of thematic analysis, where themes and subthemes are generated using an inductive approach [34, 35]. T.P. conducted preliminary coding of the data line-by-line, organized emerging codes into clusters, and developed a preliminary coding template. This template was then reviewed and refined in collaboration with J.H.. Once the coding template was finalized, T.P. applied the template to the full dataset and derived preliminary themes. These themes were discussed in collaboration with the broader author team, including the Community Advisors, for member checking, ensuring themes accurately reflected their experiences and perspectives.

## Results

### Participant characteristics

Demographics of the 12 Community Advisors participants can be found in Table 2. Participants ranged in age from 25 to 74 and were mostly women (83.3%) who were not born in Canada (58.3%).

**Table 2:** Characteristics of the f.u.n. Community Advisors who participated in the qualitative study, n = 12

Characteristic	*n* (%)
Gender	
Men	2 (16.7)
Women	10 (83.3)
Age (years)	
25–34	2 (16.7)
35–44	5 (41.7)
45–54	2 (16.7)
55–64	2 (16.7)
65–74	1 (8.3)
Ethnicity/Race	
Black	2 (16.7)
Indigenous	2 (16.7)
White	4 (33.3)
South Asian	2 (16.7)
Middle Eastern	2 (16.7)
Education	
Some high school	2 (16.7)
High school graduate	2 (16.7)
Some college	1 (8.3)
College graduate	1 (8.3)
Some university	1 (8.3)
University graduate	4 (33.3)
Postgraduate training or degree	1 (8.3)
Difficulty paying expenses	
Difficult	5 (41.7)
Very difficult	4 (33.3)
Neither easy nor difficult	2 (16.7)
I don’t know	1 (8.3)
Born in Canada	
Yes	5 (41.7)
No	7 (58.3)
Years in Canada (if not born in Canada)	
1 to 5 years	1 (14.3)
6 to 10 years	3 (42.9)
16 to 20 years	1 (14.3)
More than 20 years	2 (28.6)
Employment Status	
Employed part-time (< 35 h/week)	5 (58.3)
Unemployed	4 (33.3)
On parental leave	2 (16.7)
Retired	1 (8.3)
Marital Status	
Separated	3 (25)
Married or in domestic partnership	5 (58.3)
Divorced	2 (16.7)
Single, never married	1 (8.3)
Not listed	1 (8.3)
Parent of a child under 18	
Yes	10 (83.3)
No	2 (16.7)

## Community advisor experience results

Four themes were identified through analysis: 1) Motivation to be a Community Advisor, 2) Importance of Community Advisors to the Co-Designed Project, 3) Facilitators of Community Advisor Success, and 4) Suggestions for Improvement. A table outlining these themes and additional quotes is provided in Additional file 2 [see Additional file 2].

### Theme 1: motivation to be a Community Advisor

Participants described a range of motivations for becoming involved as Community Advisors. Within the theme “Motivation to be a Community Advisor”, three subthemes emerged including 1) social connection and community engagement, 2) helping others, 3) opportunity for skill-building.

#### Social connection and community engagement

Many Community Advisors were drawn to the role by a desire to connect with others after a period of isolation due to the COVID-19 pandemic and to meaningfully engage with their community. They emphasized the importance of building relationships and fostering a sense of belonging. One Community Advisor shared:

“*…I didn't know that this would become like my family cause I don't have family in the country. Right? So this is like, really the only place I come out to socialize is with you guys.”* (CA#12).

Another Community Advisor shared about how their own personal experiences with isolation led them to become involved,

“*I don't know anybody in Guelph at the time, I was suffering and not having enough food to eat, not knowing anybody. And all these stuff cause a lot of depression for me... I need to get out and know people… I was just lucky to hear about f.u.n.… I'm not all alone, and also when I have them it makes me happy when I see them… I think we need each other. We need to be closer with our neighbourhood. This is very important*.” (CA#11).

One Community Advisor explained how they were drawn to the role as it would inherently lead to connection by interacting with others,

*“I like [that as a] community advisor … I'll get some information and I will pass this information and I will interact with people. And I was missing that part during the COVID. I was feeling lonely. I want to be part of the community and interact with people.*” (CA#8).

#### Helping others

Community Advisors described a strong altruistic commitment to supporting other community members, particularly those who are underserved. One Community Advisor shared, “*prior to COVID I knew my neighbours were hungry. During COVID, I knew my neighbours were hungry. After COVID, I knew my neighbours were starving. So something had to be done.*” (CA#1). Another Community Advisor, putting it very directly, shared, “*I just have a need to help people, it was something that was right up my alley and I thought ‘hey I'm going to do it’. Very simple.*” (CA#3). Other Community Advisors described how their personal experiences immigrating to a new community also inspired them to be that support for others,

*“when I first came here as an immigrant I did not know much about anything and everything … when I came here I realized that there are a lot of people who are coming after me … and when I feel like I can help them in any way where now I know some of this stuff, it gives me that content feeling.*” (CA#4).

Being able to represent their own communities in this work was another reason they got involved, one Community Advisor shared,

“*As I am a member of the big community are living around here is from [country] and [country] … They need me. And I need that. I always see lack of information and a lack of resources, and I was always willing to help my community. So when f.u.n. came, I say, ‘oh, that is good opportunity to help my community.’*”(CA#7).

#### Opportunity for skill-building

Some Community Advisors expressed a strong motivation to participate in the project due to a desire to gain knowledge, learn new skills, and grow personally. One Community Advisor described that they were motivated to participate so they could learn nutrition information to support their own family’s health:

*“For me, when I first, when I see the brochure there was like nutrition that the word just I was thinking, it's like, maybe I can know more about…. And so I can be more you know, I can make more good choices for my kids. You know, because food in Canada is so different from food in [home country]. And I just wanted to be more updated.” (CA#6)*

For others, the role rekindled a long-standing interest in education or created new aspirations for self-improvement. One Community Advisor described how the experience inspired them to pursue further schooling stating, “*It’s made me want to continue my education as well, it's one of my dream.*” (CA#10). Others highlighted the value of learning from peers and the program itself: “*I also love learning*…*Every time I'm in this room I learn something new… it's not a take-take, it's a give and take” (CA#2),* and *“I have learned so much, and I'm still learning… I love it*” (CA#4).

### Theme 2: importance of Community Advisors to the co-designed project

Within the theme “Importance of Community Advisors to the co-designed project”, three subthemes emerged including 1) humanization and authenticity, 2) understanding community needs, and 3) building trust and credibility.

#### Humanization and authenticity

Many Community Advisors highlighted the importance of the Community Advisors in humanizing the project through genuine interactions that foster authenticity within the community. One Community Advisor shared,

“*I don't think I've ever been in a workplace where I haven't had to mask who I am, to some extent I think this is the first time I've ever been in this space where I'm like, almost fully, just completely myself...So it's just like we humanize the project. We are able to let our masks down, the community can let their masks down with us*.” (CA#12).

Another shared how “*most of us might live under the poverty line but we're rich with relationships*.” (CA#2), offering a glimpse into community bonds and human connection.

#### Understanding community needs

Community Advisors played a pivotal role in ensuring the f.u.n. project remained grounded in the lived realities of the community. As one Community Advisor explained, *“I feel like CAs [Community Advisors] are as important to f.u.n. as water is to life. Because if there are no CAs, there will be no f.u.n. And we cannot do anything without CAs, all of us.”* (CA#4).

Their deep understanding of community needs was repeatedly cited for the importance of the Community Advisors in the project, as one Community Advisor explained,

*“The speed, efficiency, and effectiveness of everything that we've done so far is directly correlated to the fact that we are all community members here... There's a sense of urgency because we're all in poverty... It drives the fact that we care so much.” *(CA#12).

Another shared how “*We bring community voice to the conversation, and that's where the work really begins to be done right*” (CA#2), highlighting how their involvement ensured that the project was not only informed by, but responsive to, community realities. The value of lived experience in understanding community needs was underscored by another Community Advisor who noted, “*most of us have been through it before, or we know somebody who has been. We always have some information that can help somebody.*” (CA#3).

#### Building trust and credibility

Building trust and credibility emerged as a foundational element to what Community Advisors bring to the f.u.n. project, particularly due to their shared backgrounds and lived experiences. Community Advisors emphasized that trust was more readily established when community members saw themselves reflected in those leading the work. As one Community Advisor explained, “*When they come out, if they see people that look like them or they speak their language, they feel confident…they feel they are not judged…so they feel more confident to come out*” (CA#7). Another Community Advisor described the deep skepticism many community members feel toward outside programs, shaped by past harms and systemic mistrust:

“*A lot of times I can’t tell the hand that’s reaching out to help me or the hands reaching out to hurt me…Sometimes we’d rather choose the hell we know…With a neighbour…we’ve already established that trust… we’re all in the same shit, right*?” (CA#9).

Another Community Advisor shared how Community Advisors bring a ‘boots on the ground’ approach which supports their peers in the community to seek help comfortably despite systemic mistrust,

*“It’s the boots on the ground…there’s 13 people in the community [that] people can go to for help… They don’t have to reach out to some organization and run the risk of somebody picking up a phone and calling some other organization”* (CA#1).

### Theme 3: facilitators of Community Advisor success

Within the theme “Facilitators of Community Advisor success”, three subthemes emerged including 1) respect and support among team members, 2) teamwork between Community Advisors and researchers, and 3) commitment to community.

#### Respect and support among Community Advisors

Respect and mutual support among Community Advisors were repeatedly identified as key drivers of the Community Advisors’ success in the project. Participants described the group as a safe, welcoming space where they felt valued and heard. As one Community Advisor shared, *“The respect that we all have for each other. That's the main thing, because without respect this will not grow… we all say f.u.n. love, f.u.n. family”* (CA#10). Several Community Advisors spoke about how the group dynamic helped them rediscover parts of themselves, one Community Advisor reflected *“I am coming out of my shell… I am rediscovering things. I am learning so much from these people…the space we share is…it’s beautiful.*” (CA#4). Others shared about the positive impacts on mental health and connection, describing *“This meeting time is one of the best things happening… I meet you guys and talk with you and sometimes we share meals, we share ideas. I feel like it's help, even mentally yeah, it’s affected my mental health*” (CA#8), and “*When I am here, that means I'm not alone*” (CA#11).

#### Teamwork between Community Advisors and researchers

A key factor allowing Advisors to be successful was the strong sense of teamwork between Community Advisors and the research team. Participants highlighted that this collaboration allowed them to focus on the community without being overwhelmed by logistical burdens, as one Community Advisor put it “*We could do without them [researchers], but it wouldn't be how it is… it gives us the relief and easy going… that burden is not on us.*” (CA#10). Community Advisors also emphasized the importance of being heard and respected by researchers, “*It’s also extremely important to have somebody who is listening to what we’re saying and doing their best to honour those truths… that’s not every organizer, and that’s not every project lead*” (CA#12). The spirit of teamwork was captured by another Community Advisor who reflected, “*We work together as a team rather than just a few burning themselves up because the need is so big in the community.*” (CA#2).

#### Commitment to community

A strong commitment to community was consistently present. Community Advisors emphasized that their work was driven by their commitment to addressing the needs of their community. As one Community Advisor shared,

“*We heard them. We are now doing what they asked us… Probably one of the only functional groups… where people are telling us what they would like to see and we're actually listening to them… we're not afraid to take those chances*” (CA#1).

### Theme 4: suggestions for improvement

Within the theme “Suggestions for improvement.”, two subthemes emerged including 1) wider representation and diversity among the Community Advisor team, and 2) funding and sustainability.

#### Wider representation and diversity among the Community Advisor team

Community Advisors expressed a strong desire to grow the Community Advisor team and see greater diversity and representation within the Community Advisor team, which was seen as essential to ensure that all community members feel seen, heard, and included. As one Community Advisor shared, “*We need to bring different group of people into this advisors… if they also have one voice here, they will know somebody is bringing them something.*” (CA#10). Greater language diversity was also highlighted as an area of growth, one Community Advisor expressed “*My wish is we have all kind of language in our group. We need it.*” (CA#11). These reflections underscore a vision of f.u.n. as an inclusive, evolving initiative that continues to grow alongside the community it serves.

#### Funding and sustainability

While Community Advisors expressed a strong vision for the future of f.u.n., they also voiced deep concerns about the sustainability of the work in the face of uncertain funding. There was a gap in the grant funding provided to the f.u.n. project, which was concerning to all team members, including the Community Advisors. As one Community Advisor reflected, “*It scares me that there's gonna be a gap. What happens to me? What happens to you? What happens to all of us here that still have to make ends meet…?*” (CA#2). The fear of losing momentum, relationships, and impact due to funding interruptions was a recurring theme. Community Advisors also shared a desire for autonomy and resilience in the face of these challenges, one Community Advisor explained “*We have no control over those funding cuts… I would like to have some skills to try and be able to keep it going if our funding was gone tomorrow*” (CA#2). At the same time, Community Advisors acknowledged the difficulty of aligning community-driven ideas with rigid funding structures, “*We're dreaming big not realizing that… funding is very, very siloed… We've got these big, huge dreams.*” (CA#1).

## Discussion

This study contributes to the growing body of literature on community-engaged research by examining the perceptions and experiences of community member end-users (i.e., Community Advisors) involved in a co-designed initiative aiming to increase access to nutritious foods. The findings underscore the critical role of community members in shaping the direction, relevance, and impact of the project. Their contributions, rooted in lived experience, cultural insight, and a deep commitment to their community enhanced the authenticity and responsiveness of the initiative.

Our study uncovered key motivations for participation by Community Advisors, including a desire for social connection and community engagement, a commitment to helping others, and an interest in personal and professional development through skill-building. These results mirror a previous study that found that non-researchers were motivated to engage in participatory research to enhance social connectedness, support their personal learning and development, and to make a difference in their community [36]. These motivations are not only consistent with prior research but also highlight the relational and values-driven nature of community engagement in co-designed initiatives, suggesting that Community Advisors on the f.u.n. team were deeply invested. Such intrinsic motivations likely contributed to the high level of commitment and may have enhanced both the quality of engagement and sustainability of their involvement over the length of the project. These insights have practical implications for researchers developing co-designed projects. Specifically, fostering environments that prioritize social interaction, community connection, and opportunities for skill development can enhance engagement and retention of community member end-users. Offering structured training and capacity-building opportunities may further support end-users’ personal and professional growth. Moreover, ensuring that the research is clearly aligned with community-identified goals and values, and keeping the benefit of the target community at the core of the project can strengthen trust and investment of end-users. Future co-designed research should intentionally embed these elements into study design and implementation to support meaningful and sustained community member end-user involvement.

The Community Advisors perceived that their participation brought authenticity, trust, and responsiveness to community needs to the co-designed project, which were integral to its operation and success. At the core of participatory research approaches, like co-design, is centering lived experiences. Previous research utilizing participatory approaches has found that centering lived experience fosters more authentic engagement, deepens trust, and leads to interventions that are better aligned with community priorities [37–39]. For example, McGowan et al. [18] found that co-design participants valued the opportunity to shape services based on lived experience, and that trust, shared purpose, and inclusive engagement were critical to effective co-design, though challenges such as unclear expectations and structural barriers were also noted. These findings resonate with our project, where Community Advisors ensured the project remained grounded in real-world context, values, and relationships, which likely contributed to the high acceptability of the interventions in the pilot-testing phase, where 86 to 100% of participants reported they would recommend the intervention in which they participated to another person [32]. Other participatory studies [16, 17, 40] similarly emphasize the importance of reciprocal trust, clear roles and expectations, and community leadership in shaping responsive services. These findings suggest that researchers undertaking co-design projects should prioritize true integration of lived experience throughout all phases of the research process. While co-design inherently involves active collaboration among interest holders in designing solutions to a pre-specified problem [13], it is equally important to involve community members in identifying priorities, shaping the implementation, and evaluating interventions. Structuring co-design to support authentic, reciprocal relationships and to reflect community values can enhance both the relevance and uptake of resulting interventions. Future co-designed research should consider embedding mechanisms such as iterative feedback loops and shared decision-making structures where end-users actively co-lead and shape the direction of the project to ensure responsiveness to evolving community needs.

Findings from this work also underscore explicit facilitators that allowed the Community Advisors to be successful and were closely tied to the interpersonal and structural supports embedded in the project’s design, including teamwork among both the Advisors and the researchers, as well as a strong commitment to the community across the Advisor team. These findings align with the World Health Organization’s Framework for Effective Community Engagement which emphasizes shared values of trust, mutual respect, teamwork, and a shared vision, as foundational to meaningful participation [41]. Our findings also reflect the results of Jagosh et al.’s [42] review of community-based participatory research, which identifies trust, mutual respect, and co-learning as core mechanisms driving successful engagement. Collaborative relationships described by Community Advisors also reflect what others have termed “implementation capital”, describing interpersonal and organizational supports that enable effective implementation [43]. By combining peer-based trust with dependable administrative scaffolding, the f.u.n. project cultivated an environment in which Community Advisors could thrive. In addition, the shared community commitment evident by Advisors can be conceptualized as a form of collective efficacy [44], whereby a group’s shared belief in its ability to create change enhances motivation and coordination. These insights point to the critical role of interpersonal and organizational enablers in fostering successful community engagement in participatory research and emphasize the importance of resourcing these elements in future co-designed interventions.

While Community Advisors described positive experiences overall, they also offered suggestions for improvement. One key recommendation was to further diversify the Community Advisor team, reflecting a shared desire for all cultural backgrounds in the community to be represented. Community Advisors described a willingness to help others and represent their cultural communities as motivations to participate in f.u.n.. A scoping review on the use of co-design methods with culturally and linguistically diverse communities in mental health services emphasized the importance of diversity in helping to mitigate concerns of trust, power dynamics, and communication, factors which, as described above, have been shown to significantly influence the quality of relationships in participatory research [45]. Similarly, in McGowan et al.’s work evaluating experiences of participants in the co-design of a community-based health service, participants noted a lack of diversity among the team, and echoed the importance for greater diversity as it enables participants to listen and understand diverse perspectives [18]. While these considerations are relevant in all co-design contexts, they are especially critical when working with underserved populations, as these factors impact access to and engagement with initiatives [45]. Ensuring a culturally diverse team of Community Advisors helps address these barriers and fosters more inclusive and effective participation from all relevant interest holders. Further, a notable demographic characteristic of Advisors in this study was that the majority identified as women (83.3%). This finding is consistent with previous research highlighting challenges engaging men in participatory health research [46–48]. This gender imbalance may have influenced the perspectives and priorities emphasized during the co-design process, potentially limiting the inclusion of men’s experiences or needs related to their role as a Community Advisor on this project. Future research should consider intentional strategies to engage individuals across a broader spectrum of gender identities, and integrating an intersectional analysis considering ethnicity and gender to ensure diverse perspectives are represented and equitably engaged. Advisors also expressed concerns about the sustainability of the project, particularly in relation to funding. Their experiences reflect broader critiques in the literature on peer and community-engaged research, which highlight the precarious nature of roles grounded in lived experience. These positions are frequently characterized by short-term contracts and inconsistent compensation, largely due to the limitations of grant-based funding models [49]. As researchers, we must advocate for a reorganization of research funding mechanisms to better enable sustained, equitable participation and to reduce the precarity of community-based roles in research. Their reflections also reveal not only the emotional toll of financial uncertainty, but also the tension between community-driven aspirations and the rigid, siloed nature of traditional funding structures. These insights highlight the need for funding models that are not only more stable, but also more flexible and responsive to the evolving goals of community-led initiatives. Beyond concerns about compensation, Advisors also expressed a desire for greater autonomy and additional skill-building opportunities to help sustain the initiative beyond the constraints of external funding. Providing structured training opportunities for Advisors and other community members may support this goal by fostering meaningful participation and building capacity for long-term sustainability.

Study limitations should be considered when interpreting results. First, study findings are grounded in the specific social, cultural, and geographic context of the Onward Willow neighbourhood, which may limit their transferability to other settings. Second, due to scheduling constraints of the Community Advisors, data were collected through two focus groups and one individual interview. While this flexibility enabled broader participation of the Community Advisors, the variation in format may have influenced the depth and nature of responses, particularly the opportunity for collective dialogue that is more readily facilitated in group settings. Furthermore, our findings are drawn from two focus groups and one interview with a total of 12 participants conducted at one time period, which constrains the depth and breadth of data available for analysis. Future co-design studies could benefit from inviting end-users to maintain field notes or diaries throughout the co-design process. Integrating these reflections even if facilitated from researcher provided probes alongside focus groups and interviews has been used in other co-design studies [50], and can enrich thematic interpretation and provide a more comprehensive analytic foundation. Third, although senior researchers were not present during focus groups or the interview and Community Advisors were informed that critical feedback was welcome, T.P. had established relationships with the Community Advisors, which may have introduced social desirability bias, potentially influencing how participants chose to share their experiences. Lastly, a limitation of this study is that the initial coding and theme development were conducted by a single researcher. While multi-coder approaches can enhance reliability, single-researcher coding is recognized as an acceptable method in qualitative research, particularly when combined with structured approaches such as template analysis and collaborative review [35]. While steps were taken to mitigate this limitation, including refining coding templates in collaboration, reviewing preliminary themes with the broader author team including Community Advisors through member checking, the absence of multiple independent coders remains a limitation.

## Conclusions

This study highlights the critical role of community member end-users (i.e., Community Advisors) in the co-design of food access solutions, offering valuable insights into their motivations, contributions, and the conditions that support their success. Community Advisors were driven by a desire to build social connections, help others, and develop new skills, motivations that fueled their deep engagement in the project. Their involvement brought authenticity, trust, and a nuanced understanding of community needs, reinforcing the value of embedding lived experience in participatory research, like co-design. Key facilitators of success of the co-design model included mutual respect, strong teamwork, and researcher support. At the same time, Advisors identified areas for improvement, particularly the need for broader cultural representation among the Advisors, and more sustainable funding structures. Addressing these barriers is essential for advancing more just and sustainable models of co-design and community-based research in practice for optimal long-term impact. Overall, these findings suggest that future co-designed projects should intentionally cultivate opportunities for social connection, recognize and support end-users’ skill development, and create structures that foster trust and shared ownership, as these conditions can both enhance engagement and impact.

## Supplementary Information

Supplementary Material 1.

Supplementary Material 2.

## Data Availability

The datasets analysed for the current study are not publicly available due to ethical restrictions related to the consent given by participants at the time of study commencement.

## Abbreviations

f.u.n.: food uniting neighbours
GRIPP2: Guidance for Reporting Involvement of Patients and the Public
