# Supplementary material for: Co-designing interventions to increase food access: perceptions and experiences of community member end-users
Source: Res Involv Engagem. 2025 Oct 14;11:117. doi: 10.1186/s40900-025-00788-y (PMC12522784; doi:10.1186/s40900-025-00788-y)
Supplement: Supplementary file 1 — Supplementary Material 1. [file 40900_2025_788_MOESM1_ESM.docx]

Table 1. GRIPP2 Short Form Checklist

| Section and topic | Item | Reported on page No |
| --- | --- | --- |
| 1: Aim | Report the aim of PPI in the study | 5-6 |
| 2: Methods | Provide a clear description of the methods used for PPI in the study | 7 |
| 3: Study results | Outcomes- Report the results of PPI in the study, including both positive and negative outcomes | 10-19 |
| 4: Discussion and conclusions | Outcomes – Comment on the extent to which PPI influenced the study overall. Describe positive and negative effects | 19-24 |
| 5: Reflections/critical perspective | Comment critically on the study, reflecting on things that went well and those that did not, so others can learn from this experience. | 22 |
