# Supplementary material for: Co-designing interventions to increase food access: perceptions and experiences of community member end-users
Source: Res Involv Engagem. 2025 Oct 14;11:117. doi: 10.1186/s40900-025-00788-y (PMC12522784; doi:10.1186/s40900-025-00788-y)
Supplement: Supplementary file 2 — Supplementary Material 2. [file 40900_2025_788_MOESM2_ESM.docx]

| Table 1. Illustrative quotes from participants (n=12). | | |
| --- | --- | --- |
| **Theme** | **Subtheme** | **Illustrative Quotes** |
| Motivation to be a Community Advisor | Social connection and community engagement | “I don't know anybody in Guelph at the time, I was suffering and not having enough food to eat, not knowing anybody. And all these stuff cause a lot of depression for me... I need to get out and know people… I was just lucky to hear about f.u.n… I'm not all alone, and also when I have them it makes me happy when I see them… I think we need each other. We need to be closer with our neighbourhood. This is very important.” -CA#11  “When COVID hit all of that [existing programs] went. So that's what really brought my attention to the community advisor [role]. And for the f.u.n. project and just overall working with the community on improving the barriers to social and food related items. Because I find that where there's food, there's social. Right? So those connections.” – CA#2  “I came to Guelph in 2018. So like, just before the pandemic… I knew some people because my family was here, but then, shortly after, they all moved away … We didn't have anybody to call or talk to... I joined because of money, and also because I wanted to be part of the community. I just didn't know that I would be this much a part of the community, and I didn't know that this would become like my family cause I don't have family in the country. Right? So this is like, really the only place I come out to socialize is with you guys.” – CA#12  “I like [that as a] community advisor … I'll get some information and I will pass this information and I will interact with people. And I was missing that part during the COVID. I was feeling lonely. I want to be part of the community and interact with people.” – CA#8 |
|  | Helping others | “I thought I could help…Yeah, like you know, why wouldn't I? Right? Why wouldn't I come out? I like people. especially people with less.”-CA#9  “Prior to COVID I knew my neighbours were hungry. During COVID, I knew my neighbours were hungry. After COVID, I knew my neighbours were starving. So something had to be done.” – CA#1  “I just have a need to help people, it was something that was right up my alley and I thought hey I'm going to do it. Very simple.”. – CA#3  “When I first came here as an immigrant I did not know much about anything and everything … when I came here I realized that, there are a lot of people who are coming after me … and when I feel like I can help them in any way where now I know some of this stuff, it gives me that content feeling.” - CA#4  “As I am a member of the big community are living around here is from [country] and [country] … They need me. And I need that. I always see lack of information and a lack of resources, and I was always willing to help my community. So when f.u.n came, I say, ‘oh, that is good opportunity to help my community’”. – CA#7  “When COVID came and kind of they shut everything down and when I got to know that this program is going on I was very happy to be back to the community to share more and also to learn more… this community is diverse, and people [are from] different cultures, [so they have] different needs as well… If I get to know more resources then great I can help others, … coming here also, has given me more confidence to share, and also to listen to the need of others to bring it back here too.”- CA#10  “You know, you learn stuff with people, and then you can pass it on. It makes a big difference. Yeah, it's good when you have somebody who needs help that you can help them.” – CA#3 |
|  | Opportunity for skill-building | “I also love learning. I learn all the time. Every time I'm in this room I learn something new. Every time that I'm with the community I learn something new, and so I can also bring the knowledge that I have to the table. So it's not a take-take, it's a give and take. And I really appreciate that.” – CA#2  “And also sometimes in this we feel like a little bit leader. Which I might not say I'm a leader, but I feel like I'm a little bit leader in the community, because people reach out to me to ask what is happening, is there anything going on? and I'm able to give that information, it makes me feel very proud and also grateful that people look, look up to me in the community as well…Another grateful thing is basically for like sometimes it makes me feel like I’m in the university, because I would always love to upgrade myself for school … When I talk to my family you know what I went to the meeting, and you know the table, the people on the table pull in the degrees, and that leads me for like, well, I'm on that high up position, especially the picture that we took as a group with the Masters students. And I'm inside that picture, I said to myself, I need to upgrade myself for this school to be that kind of level, and I know one day I'll get there. But this is a start that I work with Masters students, I work with the PhD student. No matter my small English, my grammar is not working, but I'm still working with it. That gives me that something joyful in me…It's made me want to continue my education as well, it's one of my dream.” – CA#10  “I am learning so much from these people like they have so so much knowledge even there. And everyone they were just talking. And they're like they know so much about like how the system is working. How like, I did not know anything like how, if I need something. how do I call? Who do I call to? You know, like I just I was just blank. I did not know anything, and until now I feel like that. I just don't know. It's a lot. But yes, I have learned so much, and I'm still learning, and that the space we share is like [this]. I love it.” – CA#4  “For me, when I first, when I see the brochure there was like nutrition that the word just I was thinking, it's like, maybe I can know more about. And I can know about Canadian food ventures. And these were the stuff. And so I can be more you know, I can make more good choices for my kids. You know, because food in Canada is so different from food in [home country]. And I just want to be more updated.” – CA#6  “But did any of you really know like going into this … that you had this leadership potential in you? - CA#1… Of course, of course, but I lost it. I lost it for a while. And then I got back to my feet in this program.” - CA#11 |
| Importance of Community Advisors | Humanization and authenticity | “I think, like a good way to put it, is like we humanize this project… Because when I think about even for myself, I don't think I've ever been in a workplace where I haven't had to mask who I am, to some extent I think this is the first time I've ever been in this space where I'm like, almost fully, just completely myself...So it's just like we humanize the project. We are able to let our masks down, the community can let their masks down with us.” – CA#12  “We live the challenges that the community is seeing daily and we bring unique points of view to the conversation, and we give community a chance. Like, if the funding is taken away tomorrow, hopefully, we'll be able to have some strong connections.” – CA#2  “Community advisors are the people who are living in the community who are facing all the hardships and maybe they are the person who knows the solutions, too, because they are they are the one who are living through all those hardships.” – CA#8  “I can say that I join another program [program name] and I am not part of that program but I attended their meetings [a] couple of times, but I feel really disconnected. They talk a lot that we did this, we did that, but they were just telling people. I feel like I couldn't connect to that program because there were only 2 or 3 people who were doing all this stuff and then telling people. But in f.u.n. I feel like I'm more connected because I'm also working and when I come to the meeting, I feel like I have a responsibility to spread this thing and come back with ideas and what people are thinking, what their response [is]. So without community advisor, it's like somebody's talking and telling the information but it's not going deep into the community. That's what most of the programs are…they are disconnected from the community.” – CA#6  “Most of us might live under the poverty line but we're rich with relationships.” - CA#2 |
|  | Understanding community needs | “Most of us have been through it before, or we know somebody who has been. We always have some information that can help somebody.” – CA#3  “…The speed, efficiency, and effectiveness of everything that we've done so far is directly correlated to the fact that we are all community members here, because if it was just a bunch of like people who come here from elsewhere or like even from here but don't socialize or don't understand the things that we go through. We wouldn't have any of the things that we have in place now, there would be no trust. There would be no effectiveness. Honestly, I think that this is the reason why…There's no sense of urgency. There's a sense of urgency because we're all in poverty, because essentially right, we're [in poverty] like most of us, anyways. So It drives the fact that we care so much.” – CA#12  “I believe that there is no f.u.n. project without community advisors. We bring community voice to the conversation, and that's where the work really begins to be done right is the community voice. We're a great link between community and the project.” - CA#2 |
|  | Building trust and credibility | “It's the boots on the ground. It's nothing for us without it…knowing that we come from the community…There's 13 people in the community [that] people can go to for help… They don't have to reach out to some organization and run the risk of somebody picking up a phone and calling some other organization right?” – CA#1  “It's the trust thing. Because they feel you are not going to lie to them because you are the same background.” – CA#10  “Even the community. When they come out, if they see people that look like them or they speak their language, they feel confident on them, they feel they are not judged, or they feel they are not misunderstanding what is going on, so they feel more confident to come out.” – CA#7  “People come into the neighbourhood with different ideas. You know, a lot of times I can't tell the hand that's reaching out to help me or the hands reaching out to hurt me, and I've been hurt so many times by people in positions of authority… it's just we shut ourselves off from letting anyone kind of just sit in our crap, you know, whatever specific brand of crap it happens to be that we're looking at if it's you know, food insecurity or income insecurity. Sometimes we'd rather choose the hell we know, rather than you know, that unknown of letting somebody else in our world to be vulnerable with …With a neighbour it's, I'm already in their backyard. I've already been on the street and maybe helped some of them in a situation where they had to let somebody help them. And so we've already established that trust…we're all in same shit right…And so it's important to have CAs in here that are in those struggles with them.” - CA#9 |
| Facilitators of Community Advisor Success | Respect and support among Community Advisors | “This meeting time is one of the best things happening… I meet you guys and talk with you and sometimes we share meals, we share ideas. I feel like it's help, even mentally yeah, it’s affected my mental health. And I didn’t know anybody.” – CA#8  “I feel like I'm more connected to the community. Otherwise I feel like in Guelph, I feel like I'm out of the world. But after joining this program I think I feel like I'm more into the community so I know what's happening around my area and where I can go if I need something I know, like I know the person I can go and talk to.” – CA#6  “When I am here, that means I'm not alone. f.u.n. family. I call you guys my f.u.n. family. I don't have any family in Ontario so my family are you.” – CA#11  “So everyone in the room like, as [CA name] said that it's like a f.u.n. family like we don't know much about each other, but gradually, slowly, we are getting to know each other, and that's beautiful. Like I am an introvert person, I just don't talk much. I don't know how to but you know, like I am coming out of my shell, too, because it's been a long time where I have been just confined to being just a mom and that's it, like nothing out of that. So I think I am feeling like I am rediscovering things. I am learning so much from these people like they have so so much knowledge…I have learned so much, and I'm still learning, and the space we share is…it's beautiful.” – CA#4  “I feel like safe here, and that's why I keep coming back. I feel safe … like for so many reasons [I come back], but like some of them would include the fact that I don't feel judged.” – CA#12  “And the respect that we all have for each other. That's the main thing, because without respect this will not grow…The respect, the connection, like we all say f.u.n. love, f.u.n. family, so if you have a family, you always get mad at each other as a family, but you keep growing, you keep building. So it's that respect that we all have for each other.” - CA#10 |
|  | Teamwork between Community Advisors and Researchers | “When I think about [PhD Student] role, [staff member] role, [MSc student] role when she was with us, [staff member] and [researcher] like, when I think of your roles and what you guys do in the background is incredible for making this project work… My whole thing is that we work together as a team rather than just a few burning themselves up because the need is so big in the community.” – CA#2  “I think that we've been AMAZING, but even if we were equally amazing and all of us were here but let's say the things we were saying were falling on deaf ears, so like if this particular human over there was not as receptive as she is (pointing to community lead), is not as organized as she is, this would not be as smooth of an operation, whereas we are like extremely important. It's also extremely important to have somebody who is listening to what we're saying and doing their best to honour those truths. And that's not every organizer, and that's not every project lead, right? So we're very, very fortunate in that sense, too.” – CA#12  “We could do without them [staff], but it wouldn't be how it is, because we'll fall apart…Doing the back work that we don't see. So you guys being behind and managing all the money and all those things. That's a lot. It gives us the relief and easy going. Like, so we knowing that this team is behind us, that burden is not on us.” – CA#10  “We're able to speak our minds and you actually listen to, so we hear information from the community and then we let the table know. And it seems like anything that the table brings, you listen, will listen.” - CA#5 |
|  | Commitment to community | “Like the CAs and the focus groups were equally important because it gave us something to sort of focus on. And again, they [community members] spoke to us. We heard them. We are now doing what they asked us. Probably one of the only functional groups in dare I say the city where we're actually doing what we're doing, where people are telling us what they would like to see and we're actually listening to them. And we're not sitting there telling them we'll get there, we'll get to it. No, we've decided that we're throwing everything at the wall, and if it sticks, we go back to it. You know, things are gonna fail. Not everything's going to be perfect. But we're not afraid to take those chances. And I think that makes a huge difference.” – CA#1  “I feel like CAs are as important to f.u.n. as water is to life. Because if there are no CAs, there will be no f.u.n. And we cannot do anything without CAs, all of us.” – CA#4  “We are the bridge for the people how they going to reach, if there is no bridge, how they going to pass? If I know the neighbour is suffering with lack of food or their kids don't have anything to wear [in] summer or winter… I saw [a] pregnant women in [the] very same clothing every day, and that is her husband's t-shirt, and I said, ‘I'm sorry, can I help you on that?’ And then she said, ‘Yeah, I don't have anything that's it’. Advisor is going to think of solving this problem as much as I could.” - CA#11 |
| Suggestions for Improvement | Wider representation and diversity among the Community Advisor team | “My wish is we have all kind of language in our group. We need it.” – CA#11  “But moving forward… it's early on, we need to bring different group of people into this advisors. Because the Nepalis and other people, they don't see anybody in this team so when they come out …’Why isn’t anybody from my country in the team?’. So, moving forward … if they also have one voice here, they will know somebody is bringing them something.” – CA#10 |
|  | Funding and sustainability | “One of the things with the co-creation, co-design model is you know, when you have funding stop, and then (hopefully) funding start up again, do you lose that great in the behind area or the community members right like, are you gonna lose that? So like it scares me that there's gonna be a gap. What happens to me? What happens to you? What happens to all of us here that still have to make ends meet and stuff like that. Right? So co-design is great, having the community with other people like another organization or a couple of organizations working together. I think it's great because it best meets the needs of the community but it really worries me when there's that gap.” – CA#2  “And we have no control over those funding cuts. So I truthfully, personally, would like to have some skills to try and be able to keep it going if our funding was gone tomorrow, right? … It scares me how quickly the funding goes.” – CA#2  “This is the only problem with the co-design model is we sometimes get tied up by somebody's imagination, right? Like we're dreaming big not realizing that you know, funding is very, very siloed…But that's the problem right? We've got these big, huge dreams.” - CA#1 |
